# ICTV Virus Taxonomy Profile: *Chrysoviridae*

**DOI:** 10.1099/jgv.0.000994

**Published:** 2017-12-18

**Authors:** Said A. Ghabrial, José R. Castón, Robert H. A. Coutts, Bradley I. Hillman, Daohong Jiang, Dae-Hyun Kim, Hiromitsu Moriyama, ICTV Report Consortium

**Affiliations:** ^1^​ Department of Plant Pathology, University of Kentucky, Lexington, KY 40546, USA; ^2^​ Department of Structure of Macromolecules Centro Nacional Biotecnología/CSIC, Campus de Cantoblanco, 28049 Madrid, Spain; ^3^​ Department of Biological and Environmental Sciences, School of Life and Medical Sciences, University of Hertfordshire, Hatfield, AL10 9AB, UK; ^4^​ Department of Plant Biology and Pathology, School of Environmental and Biological Sciences Rutgers, The State University of New Jersey, New Brunswick, NJ, USA; ^5^​ College of Plant Science and Technology, Huazhong Agricultural University, Wuhan 430070, Hubei Province, PR China; ^6^​ Division of Biological Sciences, Chonbuk National University, Dukjindong 664-14, Jeonju, Chonbuk 561-756, Republic of Korea; ^7^​ Laboratories of Molecular and Cellular Biology, Tokyo University of Agriculture and Technology, 3-5-8 Saiwaicho, Fuchu, Tokyo 183-8509, Japan

**Keywords:** *Chrysoviridae*, taxonomy, ICTV report, Penicillium chrysogenum virus, Aspergillus fumigatus chrysovirus, Helminthosporium victoriae virus 145S

## Abstract

The *Chrysoviridae* is a family of small, isometric, non-enveloped viruses (40 nm in diameter) with segmented dsRNA genomes (typically four segments). The genome segments are individually encapsidated and together comprise 11.5–12.8 kbp. The single genus *Chrysovirus* includes nine species. Chrysoviruses lack an extracellular phase to their life cycle; they are transmitted via intracellular routes within an individual during hyphal growth, in asexual or sexual spores, or between individuals via hyphal anastomosis. There are no known natural vectors for chrysoviruses. This is a summary of the International Committee on Taxonomy of Viruses (ICTV) Report on the taxonomy of the *Chrysoviridae,* which is available at www.ictv.global/report/chrysoviridae.

## Virion

**Fig. 1.**
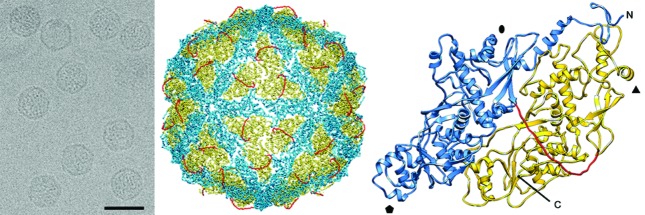
Three-dimensional cryo-EM reconstruction of Penicillium chrysogenum virus virions at a resolution of 4.1 Å. (Left) Cryo-EM image of Penicillium chrysogenum virus (scale bar, 50 nm). (Middle) Atomic model of the Penicillium chrysogenum virus capsid viewed along a twofold axis. (Right) Atomic model of a Penicillium chrysogenum virus CP (top view) showing the N-terminal domain (1–498, blue), the linker segment (499–515, red) and the C-terminal domain (516–982, yellow). Symbols indicate icosahedral symmetry axes.

Virions are isometric, non-enveloped and about 40 nm in diameter. The capsid of Penicillium chrysogenum virus comprises 60 copies of a 109 kDa polypeptide arranged on a *T*=1 icosahedral lattice (Table 1, Fig. 1). The capsid protein is formed by a repeated α-helical domain, indicative of gene duplication despite lack of sequence similarity between the two halves [1]. This domain has a fold that is conserved among dsRNA viruses [2].

## Genome

**Fig. 2.**
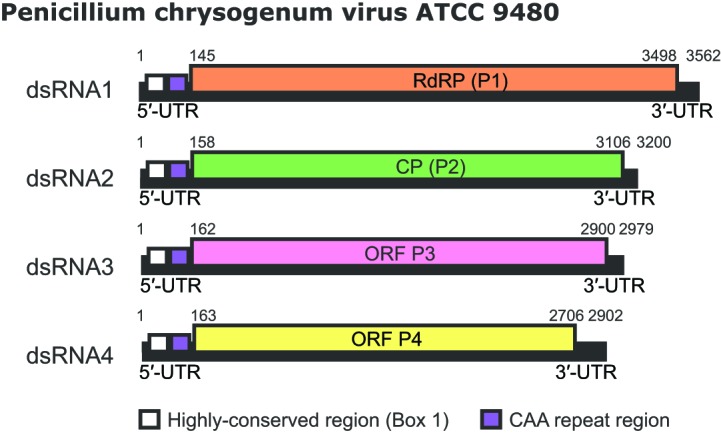
Genome organization of Penicillium chrysogenum virus isolate ATCC 9480 (PcV-ATCC9480). The genome consists of four dsRNA segments, each of which is monocistronic. The RdRP (P1) ORF (nt positions 145–3498 on dsRNA1), the CP (P2) ORF (nt positions 158–3106 on dsRNA2), the P3 ORF (nt positions 162–2900 on dsRNA3) and the P4 ORF (nt positions 163–2706 on dsRNA4) are represented by rectangular boxes.

The genome consists of four linear, separately encapsidated, dsRNA segments of 2.5–3.6 kbp [3, 4]. The largest segment, dsRNA1, codes for the virion-associated, RNA-dependent RNA polymerase (RdRP; P1) and dsRNA2 codes for the major capsid protein (CP; P2). Both dsRNA 3 and 4 encode proteins of unknown function [5]. Sequences at the 5′- and 3′-UTRs are highly conserved among the four dsRNA segments (Fig. 2). In addition to the absolutely conserved 5′- and 3′-termini, a 40–75 nt region with high sequence identity is present in the 5′-UTR of all four dsRNAs (Box 1, Fig. 2). A second region of strong sequence similarity is present immediately downstream from Box 1 and consists of a stretch of 30–50 nt containing a reiteration of the sequence ‘CAA’. The (CAA)_n_ repeats are similar to the enhancer elements present at the 5′-UTRs of tobamoviruses [6]. The N-terminal region of P3 shares high sequence similarity with the corresponding N-terminal region of RdRP (P7/P-loop domain; possibly a nucleotide triphosphate hydrase domain). P4 is a putative cysteine protease [7].

**Table 1.** Characteristics of the family *Chrysoviridae*

**Typical member:**	Penicillium chrysogenum virus ATCC 9480 (dsRNA1: AF296439; dsRNA2: AF296440; dsRNA3: AF296441; dsRNA4: AF296442), species *Penicillium chrysogenum virus,* genus *Chrysovirus*
Virion	Isometric, non-enveloped, 40 nm in diameter
Genome	A total of 11.5–12.8 kbp of dsRNA in a quadripartite genome with each segment separately encapsidated
Replication	Particles containing both dsRNA and ssRNA can be isolated from infected fungal hosts. Virions accumulate in the cytoplasm
Translation	From positive-sense transcripts of genomic dsRNAs
Host range	Fungi
Taxonomy	One genus (*Chrysovirus*) including nine species

## Replication

Replication has not been characterized in detail. Particles containing a single molecule of dsRNA, as well as particles containing both dsRNA and ssRNA, can be isolated from an infected fungal host [3]. Virions accumulate in the cytoplasm.

## Taxonomy

The family *Chrysoviridae* includes a single genus with nine species, whose members infect ascomycetous or basidiomycetous fungi.

Species demarcation criteria include nucleotide and deduced amino acid sequence data (≤70 % and ≤53 % aa sequence identity in the RdRP and CP, respectively). Chrysoviruses cause latent persistent infections in their fungal hosts. Unclassified, chrysovirus-related viruses with 3-segmented dsRNA genomes infect plants with no apparent damage [8]. Some chrysovirus-related viruses with five dsRNA genomic segments, however, cause deleterious effects in their fungal hosts [9]. blast searches using a Penicillium chrysogenum virus RdRP amino acid sequence show high sequence identity (37.6–70.2 %) to the RdRPs of members of the genus *Chrysovirus* and to related, unclassified viruses. Phylogenetic analysis based on the complete deduced amino acid sequences of RdRPs of members of the family *Chrysoviridae,* and of related, unclassified viruses with 3–5 dsRNA segments, leads to the identification of two large distinct clusters: cluster I corresponds to members of the genus *Chrysovirus* and related, unclassified viruses with three genome segments. Cluster II comprises related, unclassified viruses with four or five genome segments.

## Resources

Full ICTV Online (10th) Report: www.ictv.global/report/chrysoviridae.

## References

[1] Castón JR, Luque D, Gómez-Blanco J, Ghabrial SA (2013). Chrysovirus structure: repeated helical core as evidence of gene duplication. Adv Virus Res.

[2] Luque D, Gómez-Blanco J, Garriga D, Brilot AF, González JM (2014). Cryo-EM near-atomic structure of a dsRNA fungal virus shows ancient structural motifs preserved in the dsRNA viral lineage. Proc Natl Acad Sci USA.

[3] Buck KW, Girvan RF (1977). Comparison of the biophysical and biochemical properties of *Penicillium cyaneo*-*fulvum* virus and *Penicillium chrysogenum* virus. J Gen Virol.

[4] Jiang D, Ghabrial SA (2004). Molecular characterization of *Penicillium chrysogenum* virus: reconsideration of the taxonomy of the genus *Chrysovirus*. J Gen Virol.

[5] Ghabrial SA, Mahy BWJ, Van Regenmortel MHV (2008). Chrysoviruses. Encyclopedia of Virology.

[6] Gallie DR, Walbot V (1992). Identification of the motifs within the tobacco mosaic virus 5'-leader responsible for enhancing translation. Nucleic Acids Res.

[7] Covelli L, Coutts RH, di Serio F, Citir A, Açikgöz S (2004). Cherry chlorotic rusty spot and Amasya cherry diseases are associated with a complex pattern of mycoviral-like double-stranded RNAs. I. Characterization of a new species in the genus *Chrysovirus*. J Gen Virol.

[8] Li L, Liu J, Xu A, Wang T, Chen J (2013). Molecular characterization of a trisegmented chrysovirus isolated from the radish *Raphanus sativus*. Virus Res.

[9] Urayama S, Sakoda H, Takai R, Katoh Y, Minh Le T (2014). A dsRNA mycovirus, Magnaporthe oryzae chrysovirus 1-B, suppresses vegetative growth and development of the rice blast fungus. Virology.

