# Corrigendum: ICTV Virus Taxonomy Profile: *Chrysoviridae*

**DOI:** 10.1099/jgv.0.001032

**Published:** 2018-03-01

**Authors:** Said A. Ghabrial, José R. Castón, Robert H. A. Coutts, Bradley I. Hillman, Daohong Jiang, Dae-Hyuk Kim, Hiromitsu Moriyama, ICTV Report Consortium

**Affiliations:** ^1^​ Department of Plant Pathology, University of Kentucky, Lexington, KY 40546, USA; ^2^​ Department of Structure of Macromolecules Centro Nacional Biotecnología/CSIC, Campus de Cantoblanco 28049 Madrid, Spain; ^3^​ Department of Biological and Environmental Sciences, School of Life and Medical Sciences, University of Hertfordshire, Hatfield, AL10 9AB, UK; ^4^​ Department of Plant Biology and Pathology, School of Environmental and Biological Sciences Rutgers, The State University of New Jersey, New Brunswick, NJ, USA; ^5^​ College of Plant Science and Technology, Huazhong Agricultural University, Wuhan 430070, Hubei Province, PR China; ^6^​ Division of Biological Sciences, Chonbuk National University, Dukjindong 664-14, Jeonju, Chonbuk 561-756, Republic of Korea; ^7^​ Laboratories of Molecular and Cellular Biology, Tokyo University of Agriculture and Technology, 3-5-8 Saiwaicho, Fuchu, Tokyo 183-8509, Japan

**Keywords:** ICTV, *Chrysoviridae*

There was an error in an author’s name in the published article. The author Dae-Hyuk Kim was incorrectly given as as Dae-Hyun Kim.

Corrected author list:

Said A. Ghabrial^1​^, José R. Castón^2​^, Robert H. A. Coutts^3​^, Bradley I. Hillman^4​^, Daohong Jiang^5^, Dae-Hyuk Kim^6​^, Hiromitsu Moriyama^7​^ and ICTV Report Consortium

Additionally, in Fig. 2 the two black lines indicating the double-stranded RNA genome of each genome segment were incorrectly merged together at certain magnifications. The correct Fig. 2 is provided below.

**Fig. 2.**
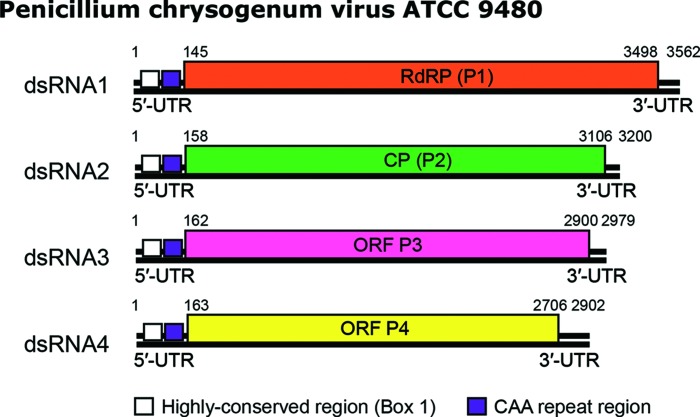
Genome organization of Penicillium chrysogenum virus isolate ATCC 9480 (PcV-ATCC9480). The genome consists of four dsRNA segments, each of which is monocistronic. The RdRP (P1) ORF (nt positions 145–3498 on dsRNA1), the CP (P2) ORF (nt positions 158–3106 on dsRNA2), the P3 ORF (nt positions 162–2900 on dsRNA3) and the P4 ORF (nt positions 163–2706 on dsRNA4) are represented by rectangular boxes.

